# A Retrospective Analysis of Intestinal Helminthic Infestations at a Tertiary Care Center in South India

**DOI:** 10.7759/cureus.72807

**Published:** 2024-10-31

**Authors:** P B Praveen Kumar, A Shanthi, Eunice Swarna Jacob

**Affiliations:** 1 Department of Microbiology, Thanjavur Medical College, Thanjavur, IND

**Keywords:** antenatal women, ascaris, deworming, hookworm, intestinal helminthic infestations

## Abstract

A parasitic worm is commonly referred to as a helminth. There are two types of helminths: Nematoda, or roundworms, and Platyhelminthes, or flatworms (flukes and tapeworms). While some are very huge, measuring more than one meter in length, many are rather large. Poor tropical and subtropical regions are the sites of the most severe helminth infections; however, some can also happen in developed nations. Less severe infestations are found throughout the planet. Food preferences, contact with vectors, climate, and cleanliness all affect an individual's risk of infection. Nearly every organ system, including the intestine, may be affected. In this study, the overall prevalence of intestinal helminthic infestations was 1.10% (n = 10), with *Ascaris lumbricoides* accounting for 0.44% (n = 4), *Ancylostoma duodenale* accounting for 0.33% (n = 3), and other helminths (*Trichuris trichiura, Enterobius vermicularis, and Taenia *species) accounting for 0.11% (n = 1) each. Over half of the world's population has historically been infected with helminths, but medical research and public health initiatives have mostly ignored them since they were thought to be non-fatal and to have little clinical importance. It is hoped that worldwide deworming programs will join the international efforts to improve the health of our planet during the next decade.

## Introduction

The most basic human need for life's existence, growth, and preservation is food. Foodborne illnesses can result from either eating food that is contaminated or from not getting enough nutrients. The spread of intestinal helminths and other foodborne parasite infestations is mostly the responsibility of food handlers [1].

The most common sources of intestinal parasites are soil-transmitted helminths (STHs), which include *Ascaris lumbricoides*, *Trichuris trichiura*, and hookworms (*Necator americanus* and *Ancylostoma duodenale*), which impact 807 million, 604 million, and 576 million individuals, respectively [1]. Soil-transmitted helminthic illnesses are reported to be endemic in India [2].

In this study, the overall prevalence of intestinal helminthic infestations was 1.10% (n = 10). Knowing the frequency of STH infections is essential for developing control plans and concentrating on areas with high endemicity for preventative treatment and better sanitation infrastructure [2].

This article was partially presented as an oral presentation at the Unicora 2023 National Conference on September 15, 2023.

## Materials and methods

Study design

This was a retrospective cross-sectional study conducted at Thanjavur Medical College, Thanjavur, India.

Study place and population

In the present study, patients who were admitted to the hospital between March 2023 and September 2024 were the subjects.

Sample collection

Patients with suspicion of intestinal helminthic infestations and other enteric pathogens were involved in this study, and on a routine basis, stool samples were received in a container with an additional spatula.

Sample rejection criteria

Stool samples received in an inappropriate container and an unlabeled or improperly labeled container were summarily rejected with proper communication with the clinical team.

Sample processing

All stool samples (n = 906) received for routine microbiological analysis were subjected to wet mounting using normal saline and Lugol’s iodine. At the clinician's request, the scotch tape method was performed to rule out pinworm infestation.

## Results

**Figure 1:**
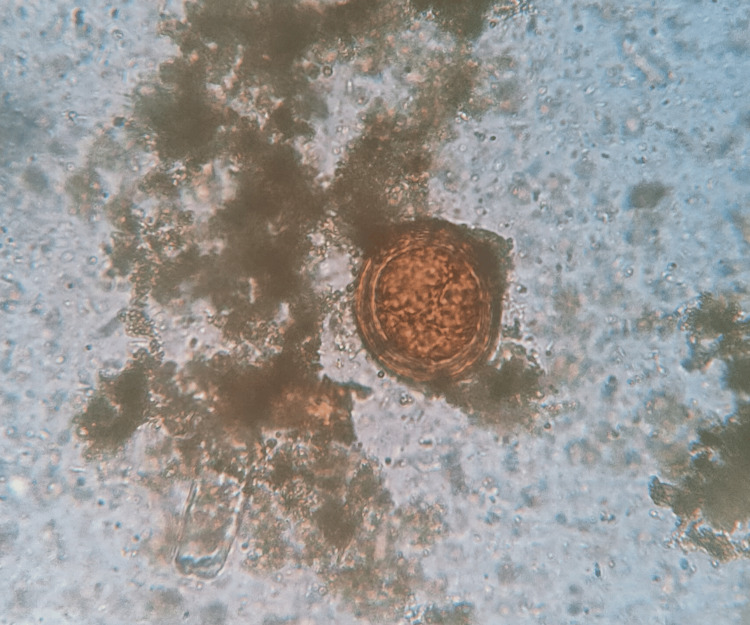
Egg of Ascaris lumbricoides Iodine wet mount (40x magnification)

**Figure 2:**
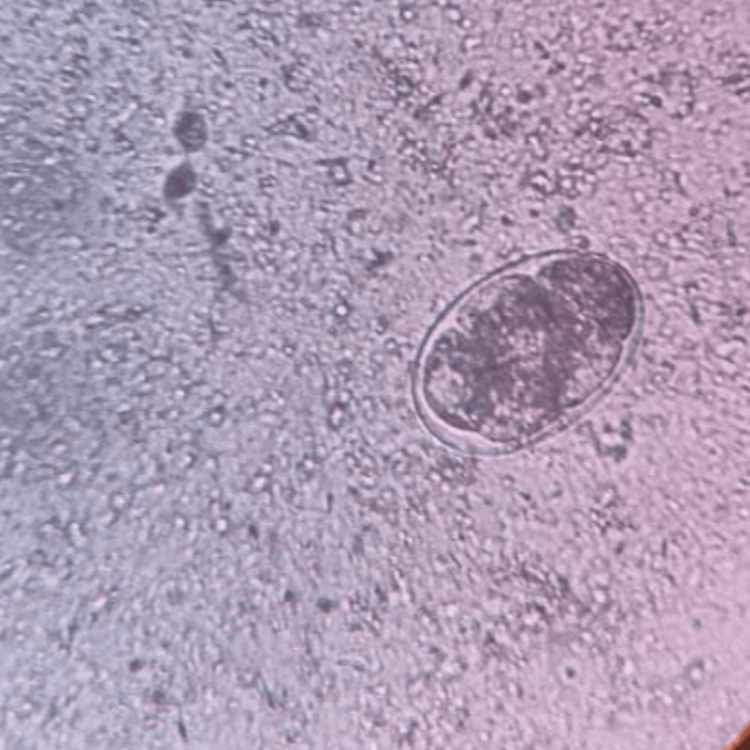
Egg of Ancylostoma duodenale Saline wet mount (40x magnification)

**Figure 3:**
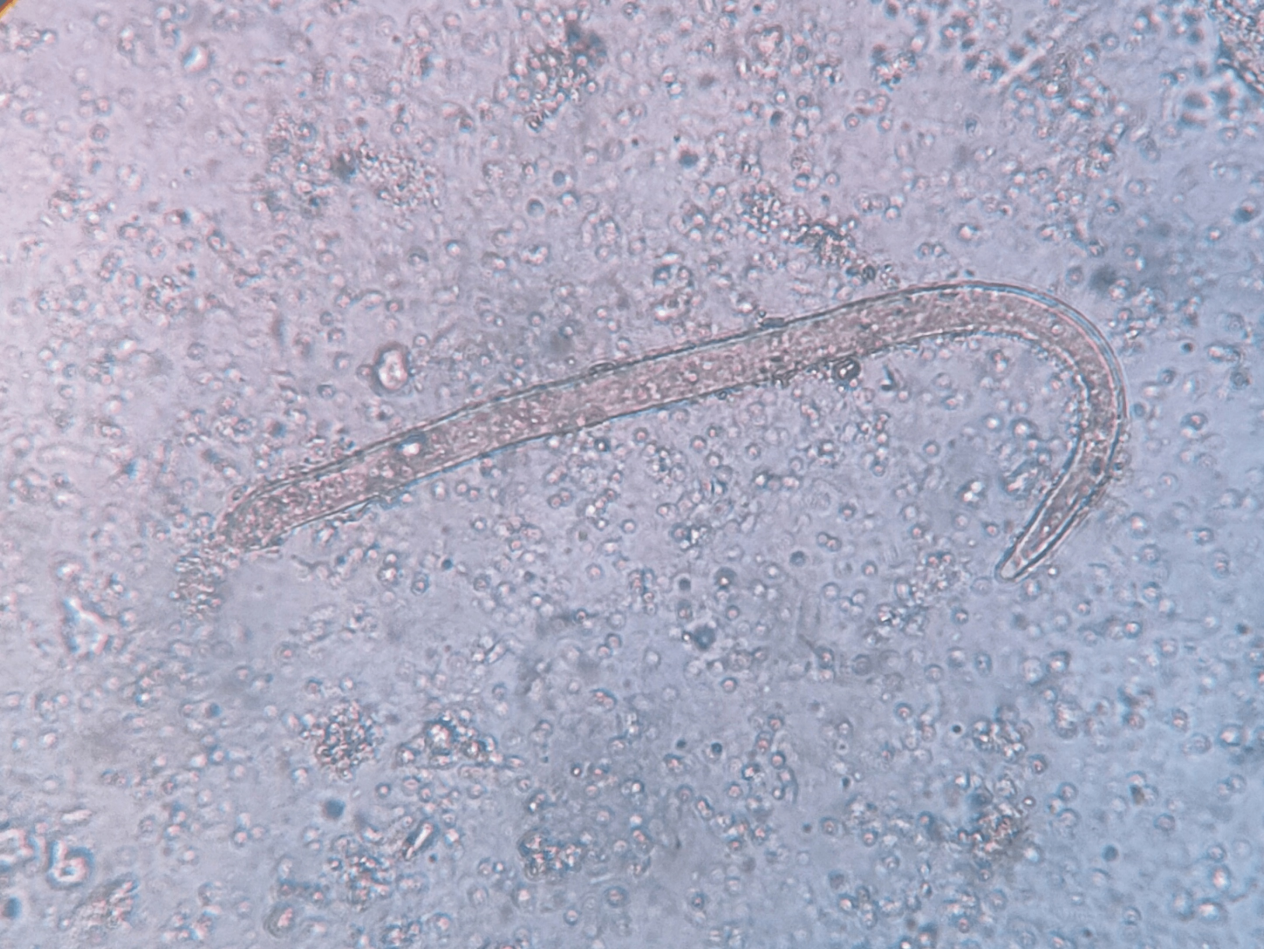
Larvae of Ancylostoma duodenale Iodine wet mount (40x magnification)

**Figure 4:**
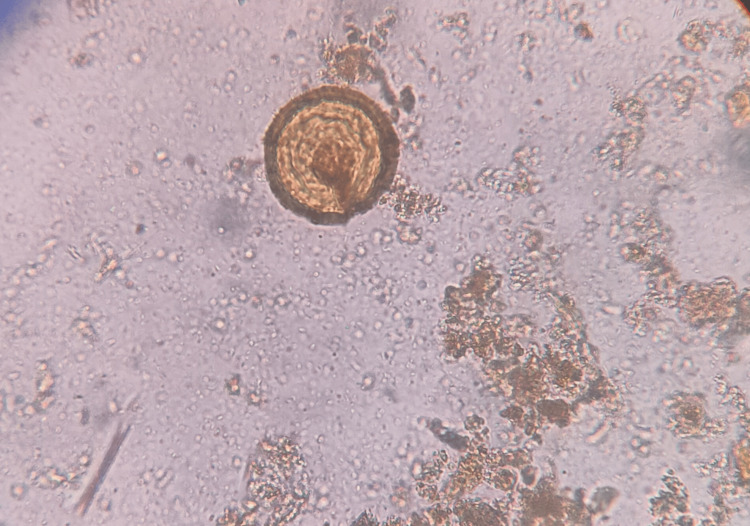
Egg of Taenia species Iodine wet mount (40x magnification)

**Figure 5:**
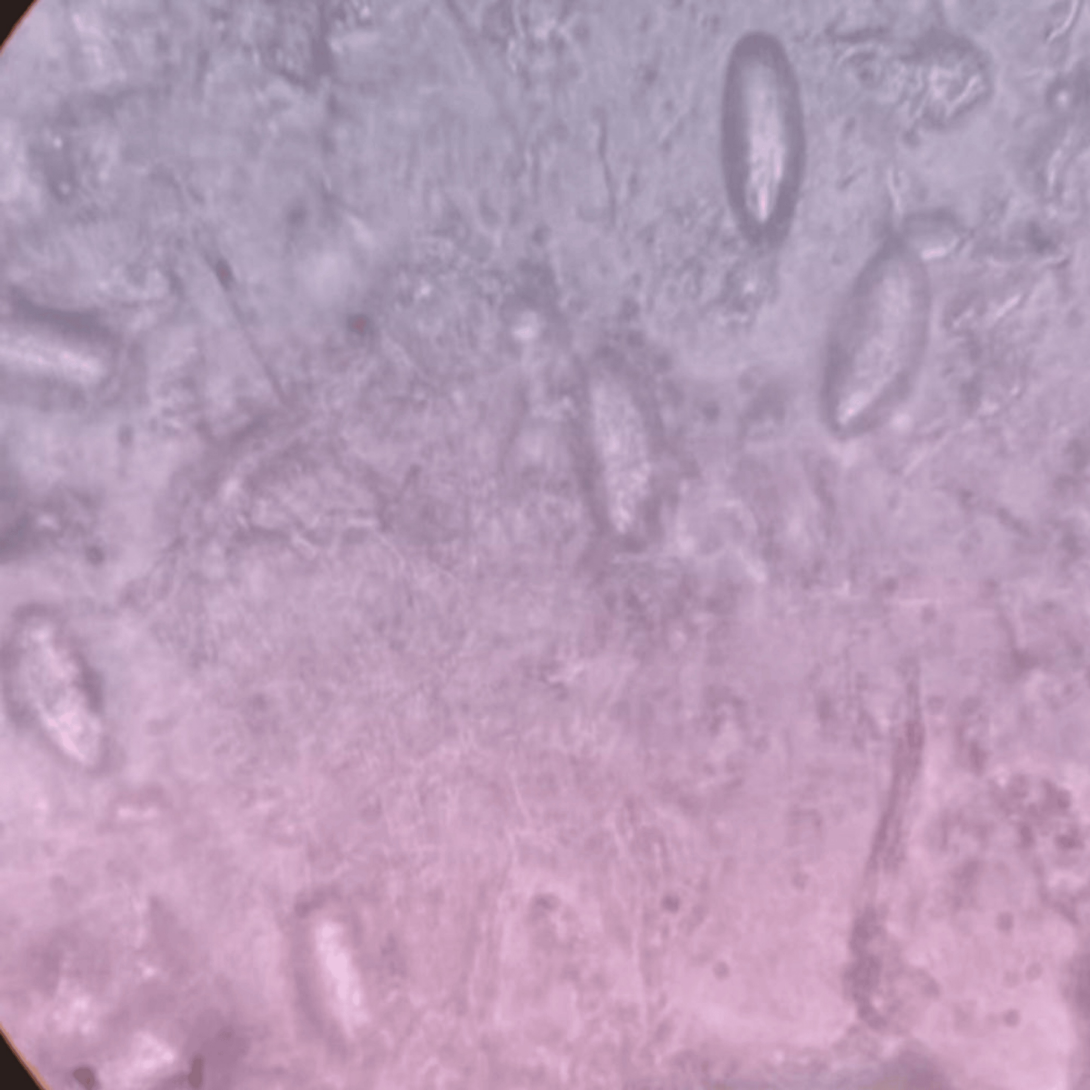
Egg of Enterobius vermicularis Scotch tape mount (40x magnification)

**Figure 6:**
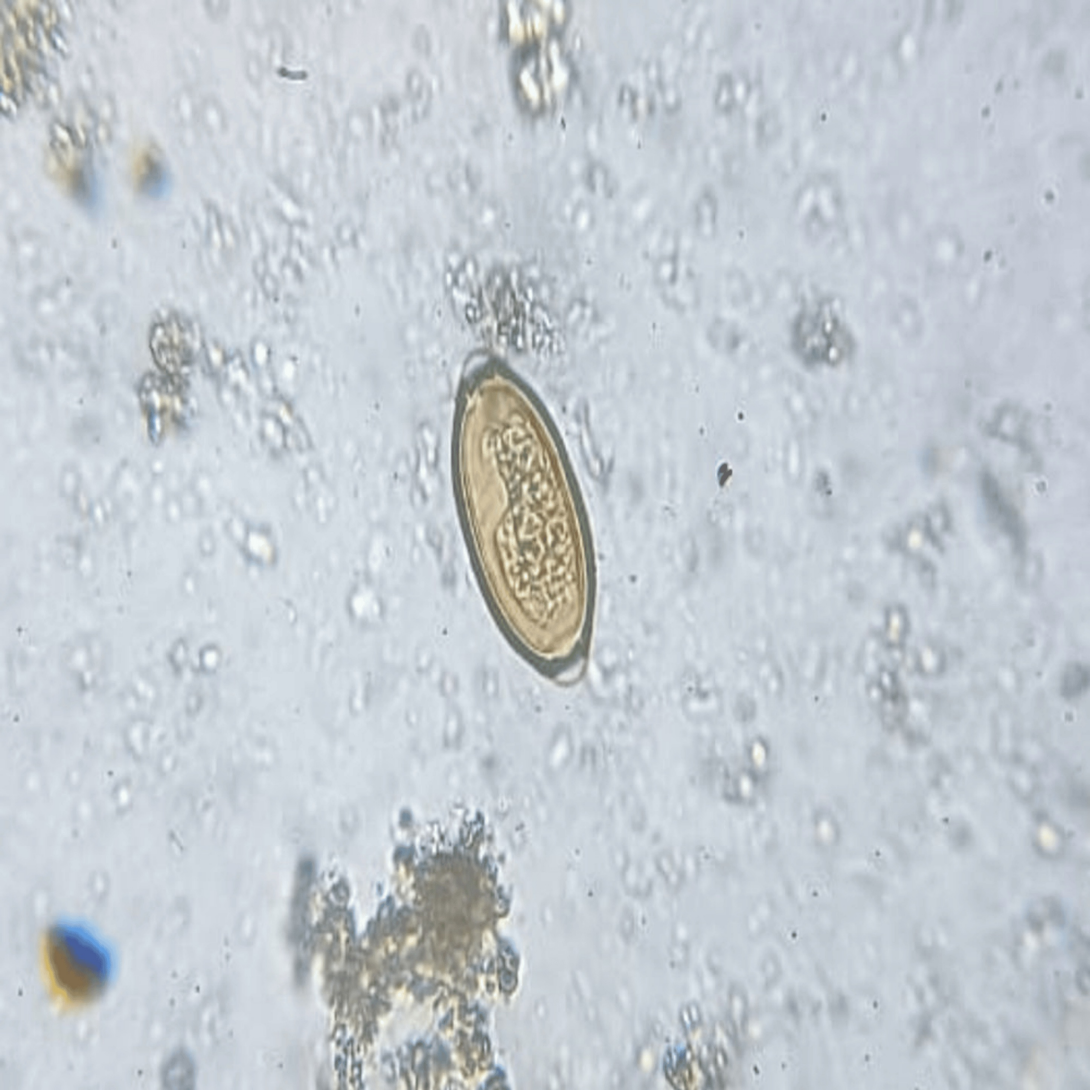
Egg of Trichuris trichiura Saline wet mount (40x magnification)

A total of 906 stool samples were received for microbiological analysis. The prevalence of intestinal helminthic infestations was found to be 1.10% (n = 10). *A. lumbricoides* (Figure 1), the most common helminth isolated, was 0.44% (n = 4), followed by *A. duodenale* (Figures 2, 3), *Taenia *species (Figure 4), *Enterobius vermicularis* (Figure 5), and *T. trichiura* (Figure 6).

After analyzing the results and residential data of the patients retrospectively, it was found that, out of 1.10% (n = 10) almost 0.88% (n = 8) of the patients residing in rural areas and using piped water as a source of drinking water. On comparing the ages of the patients involved in this study, the age group of 21 to 30 years was at the top, as shown in Table 1.

**Table 1:** Age-wise distribution of the patients

Age in range (in years)	Total number of patients (n)
≤10	11
11-20	60
21-30	343
31-40	285
41-50	112
51-60	51
≥61	44

## Discussion

The overall prevalence of intestinal helminthic infestations observed among patients in the current study was 1.10% (n = 10), with ascariasis at 0.44% (n = 4), ancylostomiasis at 0.33% (n = 3), trichuriasis at 0.11% (n = 1), enterobiasis at 0.11% (n = 1), and taeniasis at 0.11% (n = 1). In an area on the border between the districts of Vellore and Thiruvannamalai in Tamil Nadu, Southern India, a study conducted in 2013 by Kaliappan SP et al. found a prevalence of 39%, with 38% of hookworm infestation and 1.5% of ascariasis [3]. In Puducherry, a study was done among antenatal women, which revealed ascariasis (5.4%), hookworm infestation (1.8%), and strongyloidiasis (0.3%), respectively [4]. Similarly, a study done by Farook MU et al. in 2002 reported a prevalence of 23.3% with 58.82% of hookworm infestation among tribal populations of Kottoor and Achankovil areas in Kerala [5]. In Kashmir Valley, a study was done among children, which revealed the prevalence of intestinal helminths was 71.18% [6]. In Kupwara district of Kashmir, a study done by Wani SA et al. in 2007 reported a prevalence of intestinal helminths among children of 71.15% [7]. In Uttar Pradesh, a stool examination carried out by Virk KJ et al. showed a prevalence rate of 29.2% [8]. A study done by Kumar H et al. in 2014 provided a seasonal variation in intestinal helminthic infestations, with the highest prevalence recorded in the month of October and the lowest prevalence recorded in the month of January [9].

The prevalence of intestinal helminthic infestation among the patients in our study is significantly lower than the data from the previously stated studies. Overall, 1.10% (n = 10) of the participants in this study had intestinal helminthic infections. Pregnant women make up 0.77% (n = 7) of this. In Nigeria, a study on antenatal women pointed out that approximately one in five women had a helminthic infection, especially in the third trimester of pregnancy [10]. Even a study done in Ethiopia revealed a significant correlation between hookworm infestation and maternal anemia [11].

According to a study by Alemu A et al., the socioeconomic position of the patients, water availability, and environmental cleanliness may all have an impact on the variation in prevalence across various regions [12]. Poor personal hygiene practices, toilet non-use, and an uneducated population disseminate the majority of helminthic infections [13]. It has been discovered that health education techniques lower deworming costs while also raising community acceptance of deworming interventions and general health awareness [14].

Limitations and strength

This study's primary limitation is that it only used one stool examination to identify intestinal helminths, which may have understated the prevalence. Additionally, because of the retrospective cross-sectional study design, we were unable to draw conclusions about the causality of associations between different factors and helminthic infestation. Second, there is a lesser chance of finding helminths because none of the stool samples were exposed to concentration techniques. However, to the best of our knowledge, this is the first study to examine the frequency of intestinal helminthic infestations in pregnant women in Tamil Nadu.

## Conclusions

In this investigation, intestinal helminthic infections were shown to be 1.10% (n = 10) prevalent. The prevalence of *A. lumbricoides*, *A. duodenale*, *T. trichiura*, *E. vermicularis*, and *Taenia *species was 0.44% (n = 4), 0.33% (n = 3), 0.11% (n = 1), 0.11% (n = 1), and 0.11% (n = 1), respectively. Additionally, this study found that 0.77% (n = 7) of pregnant women had intestinal helminthic infestations. The results of this study should help guide the removal of intestinal helminthic infestations and the use of prophylactic chemotherapy to treat anemia that complicates pregnancy in the near future. Planning diagnostic techniques and allocating resources for the management and eradication of intestinal helminthic infestations in the Indian subcontinent will benefit from comprehensive data on the burden of disease.
